# Genetic diversity of bottlenose dolphin (*Tursiops* sp.) populations in the western North Pacific and the conservation implications

**DOI:** 10.1007/s00227-017-3232-8

**Published:** 2017-09-11

**Authors:** Ing Chen, Shin Nishida, Wei-Cheng Yang, Tomohiko Isobe, Yuko Tajima, A. Rus Hoelzel

**Affiliations:** 1 Department of Biosciences, Durham University, South Road, Durham, DH1 3LE UK; 2 Department of Life Sciences, National Cheng Kung University, 1 Da-Xue Road, East District, Tainan, 70101 Taiwan; 3 Science Education, Faculty of Education and Culture, University of Miyazaki, 1-1 Gakuen-Kibanadai-Nishi, Miyazaki, 889-2192 Japan; 4 Department of Veterinary Medicine, National Chiayi University, 580 Xinmin Road, Chiayi, 60054 Taiwan; 5 National Institute for Environmental Studies, 16-2 Onogawa, Tsukuba, 305-8506 Japan; 6 Division of Vertebrates, Department of Zoology, National Museum of Nature and Science, 4–1–1 Amakubo, Tsukuba-shi, Ibaraki, 305-0005 Japan

## Abstract

The evolutionary processes that shape patterns of diversity in highly mobile marine species are poorly understood, but important towards transferable inference on their effective conservation. In this study, bottlenose dolphins (*Tursiops* sp.) are studied to address this broader question. They exhibit remarkable geographical variation for morphology, life history, and genetic diversity, and this high level of variation has made the taxonomy of the genus controversial. A significant population structure has been reported for the most widely distributed species, the common bottlenose dolphin (*T. truncatus*), in almost all ocean basins, though no data have been available for the western North Pacific Ocean (WNP). The genetic diversity of bottlenose dolphins in the WNP was investigated based on 20 microsatellite and one mitochondrial DNA markers for samples collected from Taiwanese, Japanese, and Philippine waters (9°–39°N, 120°–140°E) during 1986–2012. The results indicated that there are at least four genetically differentiated populations of common bottlenose dolphins in the western and central North Pacific Ocean. The pattern of differentiation appears to correspond to habitat types, resembling results seen in other populations of the same species. Our analyses also showed that there was no evident gene flow between the two “sister species”, the common bottlenose dolphins, and the Indo-Pacific bottlenose dolphins (*T. aduncus*) occurring sympatrically in our study region.

**Electronic supplementary material:**

The online version of this article (doi:10.1007/s00227-017-3232-8) contains supplementary material, which is available to authorized users.

## Introduction

A wildlife management unit is usually defined by the significance of morphological, genetic, or demographic differences among populations, often associated with geographic barriers or distance (e.g. Allendorf and Luikart 2006). Identifying management units is imperative in wildlife conservation, as it assists the preservation of intra-species diversity and the species’ future adaptive potential. Some oceanic dolphin species show an unexpected level of population structure, given their capacity for extensive dispersion and the lack of obvious geographic barriers (Hoelzel 2009). The bottlenose dolphin (*Tursiops* sp.) has provided a number of classic examples regarding the parapatric or sympatric distribution of differentiated populations or species (e.g., see Moura et al. 2013). Here, we investigate bottlenose dolphin populations in a region where there is ongoing impact due to bycatch and drive fisheries, and, therefore, a pressing need for conservation management.

Bottlenose dolphins are widely distributed in the world’s tropical-to-temperate marine environment, including along the coasts of all major continents and many oceanic islands, over shallow offshore banks or sandbars, and in pelagic open waters (Rice 1998). There is considerable geographical variation in bottlenose dolphin skeletal morphology, life history, and genetic diversity, which makes the taxonomy of the genus controversial (Rice 1998; Wells and Scott 2009; Charlton-Robb et al. 2015). In our study region in the western North Pacific Ocean (WNP), two species of dolphins in the genus *Tursiops* have been recognized: the Indo-Pacific bottlenose dolphin (*T. aduncus*; hereinafter IPBD) and the common bottlenose dolphin (*T. truncatus*; hereinafter CBD). These two species are distributed parapatrically, or even sympatrically in particular areas. The distribution of IPBD is chiefly in the coastal waters of warm-temperate-to-tropical Indo-Pacific regions from southern Japan to western South Africa and southeastern Australia, where the water depth is always less than 200 m (Wang and Yang 2009). The distribution of CBD in the WNP, on the other hand, ranges from the southern Okhotsk Sea to the South China Sea and to Hawaiian waters, in both coastal and pelagic habitats (Miyashita 1993; Rice 1998; Wells and Scott 2009). The distribution range of these two species overlaps from the East China Sea and Taiwan Strait to the South China Sea (Zhou and Qian 1985; Wang et al. 1999, 2000; Yang et al. 2005). Although the broader taxonomy of the genus remains unresolved, the alpha taxonomy of IPBD and CBD is well supported (LeDuc et al. 1999; Wang et al. 1999, 2000; Hale et al. 2000; Kemper 2004; Natoli et al. 2004; Yang et al. 2005; Kurihara and Oda 2007; Moura et al. 2013).

Within the CBD species, a significant differentiation between coastal and offshore populations has been reported from various locations, including the western North Atlantic Ocean (Hoelzel et al. 1998; Kingston and Rosel 2004), the eastern North Atlantic Ocean (Louis et al. 2014a), and the eastern North Pacific Ocean (Lowther-Thieleking et al. 2015). The population structure of CBD can also be defined on a finer regional scale, such as within the Gulf of Mexico (Sellas et al. 2005; Richards et al. 2013), Northern Bahamas (Parsons et al. 2006), west coasts of the United States (Rosel et al. 2009), the waters around New Zealand (Tezanos-Pinto et al. 2009), Ireland (Mirimin et al. 2011), Hawaiian Islands (Martien et al. 2012), and the Adriatic Sea (Gaspari et al. 2015a).

Two recent papers analysed mitochondrial DNA (mtDNA) control region sequence data for CBD and IPBD with samples from the western South Pacific Ocean and hypothesised that the coastal ecotype of CBD is lacking in the Indo-western Pacific Ocean and that this is because the coastal habitat has been occupied by IPBD (Tezanos-Pinto et al. 2009; Oremus et al. 2015a). However, not all coastal and pelagic CBD lineages are reciprocally monophyletic (Moura et al. 2013), sometimes likely due to incomplete lineage sorting (Segura et al. 2006; Lowther-Thieleking et al. 2015). Therefore, this assessment should be confirmed using nuclear markers.

Miyashita (1993) proposed a three-stock structure for CBD in the WNP (for the waters off eastern Japan) based on 8-year transect line survey data: a Japanese coastal population (from the east coasts of Japan to the west of 142°E), a Japanese offshore population (between 30° and 42°N and from the east of 145°E to the antimeridian), and a southern offshore population (between 23° and 30°N, and between 127°E and the antimeridian). However, this three-stock hypothesis has yet to be tested using molecular markers. Kita et al. (2013) sequenced a group of 165 CBD culled in a drive fishery hunt in Japan for 402 bp mtDNA control region and compared these against published sequences worldwide (using 290 bp). They report that these dolphins were “related more closely to oceanic types from Chinese waters than other geographic regions” (p. 476). The study was unfortunately unable to provide further insights into the population structure of CBD in the WNP.

For IPBD, it has been proposed that there are at least six populations in Japanese waters (Amano 2007; Brownell and Funahashi 2013). Kakuda et al. (2002) studied the genetic structure of IPBD from Mikura Island (about 200 km south of Tokyo) using mtDNA control region sequences and concluded that the dolphins were genetically similar to the IPBD in Taiwanese waters. Hayano (2013) used the same genetic marker and reported a clear population differentiation among Mikura, Amakusa, Amami, and Ogasawa Islands. The residency of Amakusa, Mikura Island, and Kagoshima Bay populations has been proposed based on photo-identification records (Shirakihara et al. 2002; Kogi et al. 2004; Nanbu et al. 2006). A significant geographic vocalization variation is found among dolphin populations around Amakusa, Mikura, and Ogasawa Islands (Morisaka et al. 2005). In Taiwanese waters, the distribution of IPBD is seemly discontinuous: current field observations and records of fishery interactions showed that this species aggregates around the Penghu archipelago (in the Taiwan Strait, west of Taiwan), and the coastal waters off Kengting, southeast of Taiwan (Wang et al. 1999; Wang 2000).

Both CBD and IPBD are affected by multiple anthropogenic threats, such as small-scale whaling and negative fishery interactions in this WNP study region (Perrin et al. 2005; Kasuya 2007; Young and Iudicello 2007; Robards and Reeves 2011). There were more than 26,000 bottlenose dolphins caught in Japanese waters during 1972–2008 (Kasuya 2011), and about 1700 bottlenose dolphins are incidentally killed in human fisheries in the western-central Pacific Ocean every year (Young and Iudicello 2007). The aim of this study is to promote the more effective conservation of these highly mobile marine species through a better understanding of the pattern and origin of population structure, and the relevant processes. We focus on the CBD species in the WNP to help assess the impact of human disturbance, since this species is a common target in the dolphin drive fishery (Kasuya 2007; Oremus et al. 2015b).

## Materials and methods

### Tissue sample collection and genomic DNA preparation

**Fig. 1:**
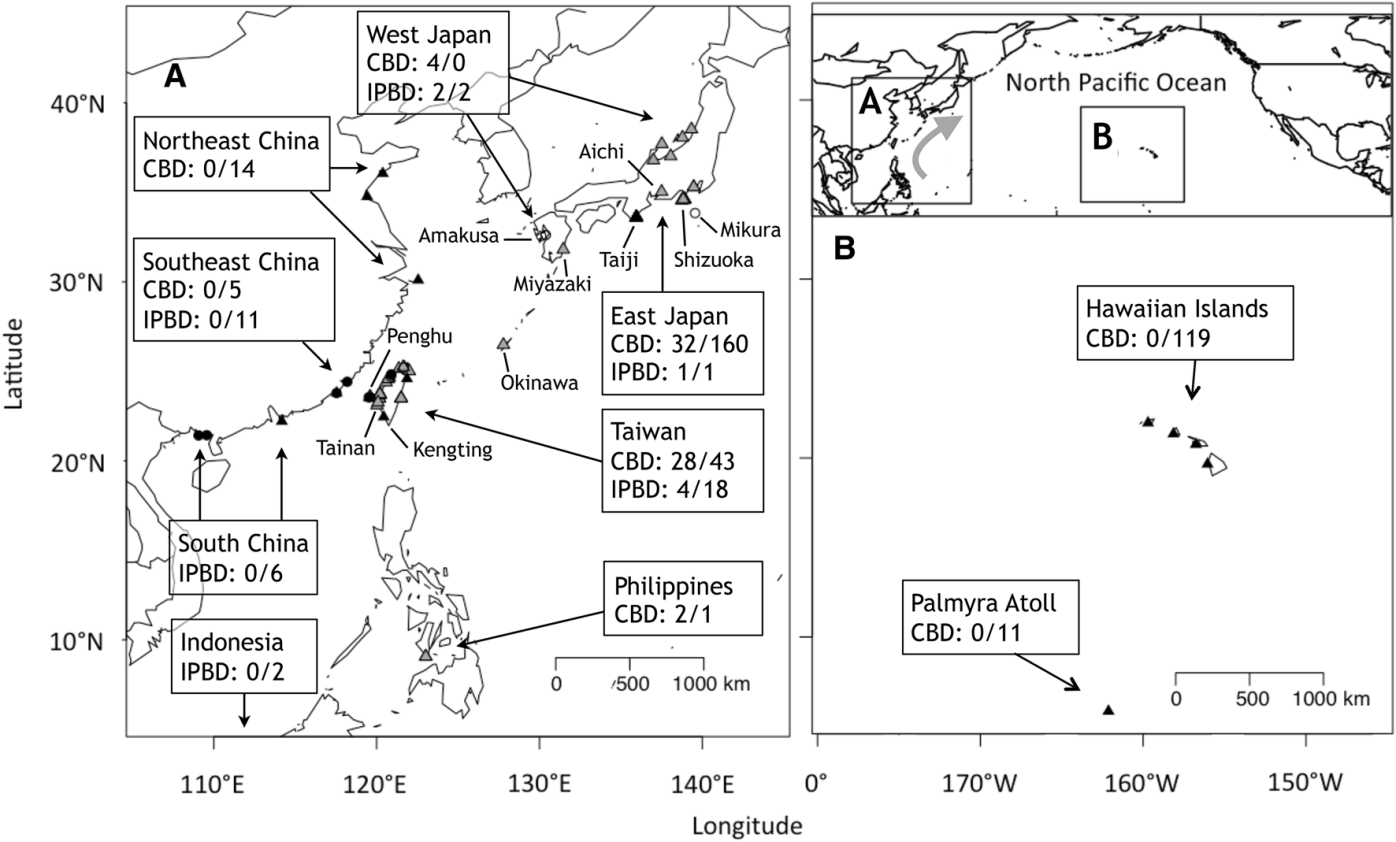
Sampling locations are provided for the Indo-Pacific bottlenose dolphins (IPBD; *open circle*) and common bottlenose dolphins (CBD; *grey triangle*) examined in this study, and the IPBD (*solid circle*) and CBD (*solid triangle*) mitochondrial DNA sequences acquired from GenBank. The *numbers* indicate the sample size for microsatellite/mtDNA data. Note that the sampling locations of the two IPBD samples from Indonesia are unknown, therefore, not indicated (see Wang et al. 1999). The *grey arrow* in *upper right panel* indicates the flow of Kuroshio current

Sixty-six CBD and seven IPBD tissue samples collected from various locations in Japan, Taiwan, and the Philippines were included in this study (detailed locations and numbers shown in Fig. 1 with further details provided in supplementary Table S1). Species identity was acquired from the archives and verified by our genetic assessments. For CBD samples, each was assigned to one of the four putative populations based on its sampling location (i.e., West Japan, East Japan, Taiwan, and the Philippines; Fig. 1a; Table S1). The origin of the 14 samples collected from Japanese aquariums was unknown, but assigned to the East Japan group based on our factorial correspondence analysis (FCA) result (see below). For IPBD, the *a prior* population assignment was based on sampling location (Fig. 1a; Table S1). For a set of 15 samples from a drive fishery in eastern Japan, we note that none of these samples were collected to support this work. Our use of archived materials derived from those activities is not meant as an endorsement, but rather as a means to contribute to the provision of data critical to the effective conservation of these populations.

Genomic DNA was isolated and purified by a standard proteinase-K digestion/phenol–chloroform extraction protocol (Sambrook et al. 1989), and preserved in TE buffer (10 mM Tris–HCl, 0.1 mM EDTA, pH7.4). The Philippine samples were provided as extracted genomic DNA by the Southwest Fisheries Science Center, National Oceanic and Atmospheric Administration (USA).

### DNA fragment amplification and genotyping

We examined 20 microsatellite loci and a 388 bp mtDNA control region sequence that have been conventionally used and validated in other bottlenose dolphin genetic studies (Shinohara et al. 1997; Hoelzel et al. 1998; Krützen et al. 2001; Natoli et al. 2004; Mirimin et al. 2011). The microsatellite loci and associated annealing temperatures are given in Table S2. Polymerase chain reaction (PCR) reagents were in 20 or 10 μl using GoTaq^®^ Taq DNA polymerase (Promega) or multiplex polymerase (Multiplex PCR Kit, Qiagen), respectively, cycled at 95 °C for 120 s (15 m for multiplex polymerase), followed by 35 cycles of 40 s at 94 °C, 40 s at the best annealing temperature of the locus, and 70 s at 72 °C, and a post-extension at 72 °C for 10 min. Fragments were visualised on an Applied Biosystems 3730 DNA Analyser, and the allele size was determined by an internal standard marker (Genescan-500 ROX, Applied Biosystems) and visualised in Peak Scanner v.1 (Applied Biosystems). Every locus in each sample was examined at least twice and the scores blind confirmed by a second person.

The mtDNA sequences were amplified using primers designed to amplify cetacean mtDNA control region (Thr and Phe primers from Hoelzel et al. 1991). The PCR reactions were in 20 μl cycling at 95 °C for 120 s, followed by 35 cycles of 40 s at 94 °C, 40 s at 50 °C, and 70 s at 72 °C, and a post-extension at 72 °C for 10 min. The amplified mtDNA fragments were purified using QIAquick^®^ PCR Purification Kit (Qiagen) and then sequenced on an Applied Biosystems 3730 DNA Analyser. All sequencing results were visualised in FinchTV (PerkinElmer) and manually corrected using MEGA 5.05 (Tamura et al. 2011).

### Microsatellite data analysis

#### Genetic diversity and differentiation

We used Arlequin 3.5.1 (Excoffier and Lischer 2010) to examine linkage disequilibrium (LD), observed heterozygosity (H_O_), and expected heterozygosity (H_E_), and to assess the significance of any deviation from Hardy–Weinberg equilibrium (HWE) using Fisher’s exact test and Markov chain method (number of steps in Markov chain, 1000,000; number of dememorization steps, 100,000). The inbreeding coefficient (F_IS_) for each locus in each putative population was estimated using FSTAT 2.9.3.2 (Goudet 2002). We also used Arlequin to assess the degree of population differentiation by *F*_ST_ (Wright 1951) and *R*_ST_ (Slatkin 1995), only for populations with a sufficient sample size (East Japan; *n* = 32 and Taiwan; *n* = 28). We used a non-parametric permutation approach with 10,000 permutations to assess the statistical significance (*p* < 0.05 after Bonferonni correction).

Genetic structure was also investigated by FCA implemented in Genetix 4.0 (Belkhir et al. 2004), and using the Bayesian assignment method implemented in STRUCTURE (Pritchard et al. 2000). FCA was run both with and without referencing individuals to a population centre (the “sur population” option). In STRUCTURE, we used six independent runs for each value of *K* assuming admixture and applying a burn-in length of 100,000 and a length of simulation of 1,000,000 repeats. The analysis was undertaken with and without using the “LOCPRIOR” function. Delta *K* (Δ*K*) reflecting the highest hierarchical level was determined by the Evanno method implemented in Structure Harvester (Earl and von Holdt 2012), and the result was optimized using CLUMPP (Jakobsson and Rosenberg 2007) and DISTRUCT (Rosenberg 2004). Once *K* was determined, we used the USEPOPINFO option to search for potential hybrids or descents of hybrids following the method described in Martien et al. (2012).

We also used the R package Geneland (Guillot et al. 2005) to assess the population structure in a spatial context. Because the program requires information of precise spatial coordinates for each genotyped individual, those CBD samples with ambiguous sampling locations were excluded for this analysis. In particular, the Japanese samples collected from the aquariums and the Taiwanese samples confiscated in the fish markets were excluded. We conducted the analysis using the procedure described in Fontaine et al. (2007), but the number of clusters (*K*) was set to vary from 1 to 6 clusters, and the maximum rate of Poisson process fixed to 41 (number of samples), uncertainty attached to spatial coordinates was fixed to 100 km, maximum number of nuclei in the Poisson–Voronoi tessellation was fixed to 123, and the posterior probabilities of population membership for each individual and each pixel of the spatial domain were calculated with a burn-in of 100 iterations and a spatial domain of 151 pixels along the *X*-axis and 250 along the *Y*-axis.

#### CBD population dynamics in the WNP

The effective population size (*N*_e_) and long-term gene flow (the number of migrants per generation; *N*_e_ *m*) were estimated using maximum-likelihood coalescent methods implemented in MIGRATE version 3.6.6 (Beerli and Felsenstein 1999, 2001). The settings were after Martien et al. (2012), but we used a heating scheme and repeated the analysis five times. An approximate *N*_e_ was calculated as the *N*_e_ *μ* divided by an average expected microsatellite mutation rate, *μ* = 5 × 10^−4^ (Whitaker et al. 2003; Hoelzel et al. 2007; Hollatz et al. 2011). The ratio of effective to census population size (*N*_e_ *N* ^−1^; Frankham 1995) was calculated using published estimates of the census population sizes for CBD populations found in Japanese waters. We used the census population size (*N*) estimated for the “Japanese Coastal” population (*N* = 37,000; Miyashita 1993) for the “East Coast Cluster” (see below), and the *N* estimated for the CBD in southwestern Japanese waters (*N* = 35,000; Kasuya 2011) for the “West Coast Cluster”. We used GeneClass2 to search for potential first-generation migrants (Piry et al. 2004) and computed the likelihood using the algorithm described in Paetkau et al. (2004), with a frequency-based method (Paetkau et al. 1995). The probability was estimated using MCMC resampling of 1000 individuals and the type I error was set to 0.01. Tests for sex-biased dispersal were implemented in FSTAT (Goudet et al. 2002). This is based on differences between the sexes for statistics associated with mean and variance of assignment indices, *F*_IS_, *F*_ST_, relatedness, *H*_O_, and within-group gene diversity (*H*_S_) with *t* tests using 1000 permutations.

### Mitochondrial DNA data analyses

#### Multi-region network

Published mtDNA control region sequences for both CBD and IPBD from the same or adjacent regions, i.e., Taiwan and southeastern China (Wang et al. 1999; Yang et al. 2005), Japan (Kita et al. 2013), northeastern China (Yang et al. 2005), and Hawaiian Islands and Palmyra Atoll (Martien et al. 2012), were acquired from GenBank (see Table S3). To address strong kin bias for our samples, one individual from all recognized parent–offspring pairs was discarded. In the published sequences, the pedigree relationship among individuals for Japanese, Hawaiian, and Palmyra Atoll samples was well documented (Martien et al. 2012; Kita et al. 2013), but the kinship information for Chinese samples was not available (Wang et al. 1999; Yang et al. 2005). We assumed that there was no parent–offspring pair sampled in the Chinese samples, because (1) they were collected in independent strandings or occasional fishery interaction events, (2) only a few individuals shared the same haplotype, and more importantly, (3) those samples sharing the same haplotype were not collected at the same time or location. We aligned the sequences together with ours in MEGA and obtained a consensus sequence (388 bp) from which we generated a median joining network using the program POPART (Leigh and Bryant 2015). We then assigned these published mtDNA sequences together with ours to six putative populations based on their sampling geography; that is, Japan, Northeast China (including Zhoushan, Qingdao, and Lianyunggang), Southeast China (including Dongshan, Taiwan, Hong Kong, the Philippines), South China (Beihai), Indonesia, Hawaiian Islands, and Palmyra Atoll (Fig. 1; Table S3).

#### Genetic diversity and tests for population expansion history

We used DnaSP v5 (Librado and Rozas 2009) to identify the haplotypes and estimate the nucleotide diversity (*π*) and haplotype diversity (*h*) for each putative population, as well as for the overall species. The neutrality tests Tajima’s D (Tajima 1989) and Fu’s Fs (Fu 1997) were estimated using DnaSP to look for signals that could be interpreted as either selection or population expansion. Mismatch distributions generated in Arlequin were produced to test for population expansion signals (Rogers and Harpending 1992; Schneider and Excoffier 1999; Excoffier 2004; Ray et al. 2003). The confidence intervals of the estimates were obtained under 10,000 bootstrap simulations of an instantaneous expansion under a coalescent framework. The sum of square deviations (SSD) between the observed and the expected mismatch and the raggedness index (*r*) of the observed distribution were calculated and tested to evaluate fit to models (Harpending 1994; Schneider and Excoffier 1999).

#### CBD population structure in the western and central North Pacific Ocean

Global tests of genetic differentiation among samples, as well as a differentiation test between all pairs of putative populations, were assessed using a Fisher’s exact test (Raymond and Rousset 1995) implemented in Arlequin, using 10,000 permutations. Pairwise *F*_ST_ and Φ_ST_ between all pairs of putative populations were calculated and tested for significance using Arlequin. The significance level was set as *p* < 0.05.

We used MrBayes 3.2 (Ronquist et al. 2012) to reconstruct the phylogeny of all CBD haplotypes using a Bayesian Markov Chain Monte Carlo (MCMC) analysis. The evolutionary model for the test was determined by jModelTest 2.1.5 (Darriba et al. 2012); the sampling increment was set at 100 and diagnostics at every 1000 generations; at least 900,000 generations were simulated to generate the consensus tree. The final consensus tree was visualized and edited for optimal display in FigTree v.1.4 (http://tree.bio.ed.ac.uk/software/figtree/).

## Results

### CBD microsatellite locus diversity and differentiation

All samples were successfully genotyped, and the missing data rate was less than 5% for all loci and samples. No significant LD or deviation from HWE was detected. Summary statistics are shown in Table 1 for each putative population (see Table S4 for the information by locus). The *F*_ST_ between East Japan and Taiwan was 0.013 and significantly greater than zero (*p* < 0.01); while *R*_ST_ was 0.055, but not significant (*p* = 0.068 ± 0.002). As expected based on simulation studies (e.g., Latch et al. 2006), the magnitude of differentiation was too small for clear detection by STRUCTURE. However, although LnP(*K*) indicated *K* = 1, Δ*K* = 2 when the LOCPRIOR function is applied, and then differentiation between East Japan and Taiwan/West Japan could be detected (Fig. S1).

**Table 1:** For common bottlenose dolphins (CBD) and Indo-Pacific bottlenose dolphins (IPBD), the number of alleles, expected heterozygosity (H_E_), observed heterozygosity (H_O_), allelic richness (AR), and inbreeding coefficient (*F*_IS_) averaged across loci within populations (Mean ± SD)

Population	*n*	No. of alleles	*H*_E_	*H*_O_	AR	*F*_IS_
CBD						
Taiwan	28	7.100 ± 2.882	0.715 ± 0.193	0.697 ± 0.192	1.715	0.025
East Japan	32	7.350 ± 3.558	0.715 ± 0.172	0.702 ± 0.189	1.715	0.019
West Japan	4	3.750 ± 1.517	0.695 ± 0.225	0.684 ± 0.248	1.661	0.019
Philippines	2	2.650 ± 0.875	0.769 ± 0.173	0.694 ± 0.349	1.692	0.138
All samples	66	8.400 ± 3.789	0.717 ± 0.179	0.695 ± 0.176	1.717	
IPBD						
Taiwan	4	3.111 ± 0.963	0.651 ± 0.134	0.625 ± 0.196	1.586	0.046
Amakusa	2	2.385 ± 0.506	0.641 ± 0.165	0.615 ± 0.300	1.417	0.059
Mikura Island	1	NA	NA	NA	NA	NA
All samples	7	3.350 ± 1.137	0.601 ± 0.190	0.549 ± 0.180	1.601	

See Table S4 for the estimates by locus within each population

**Fig. 2:**
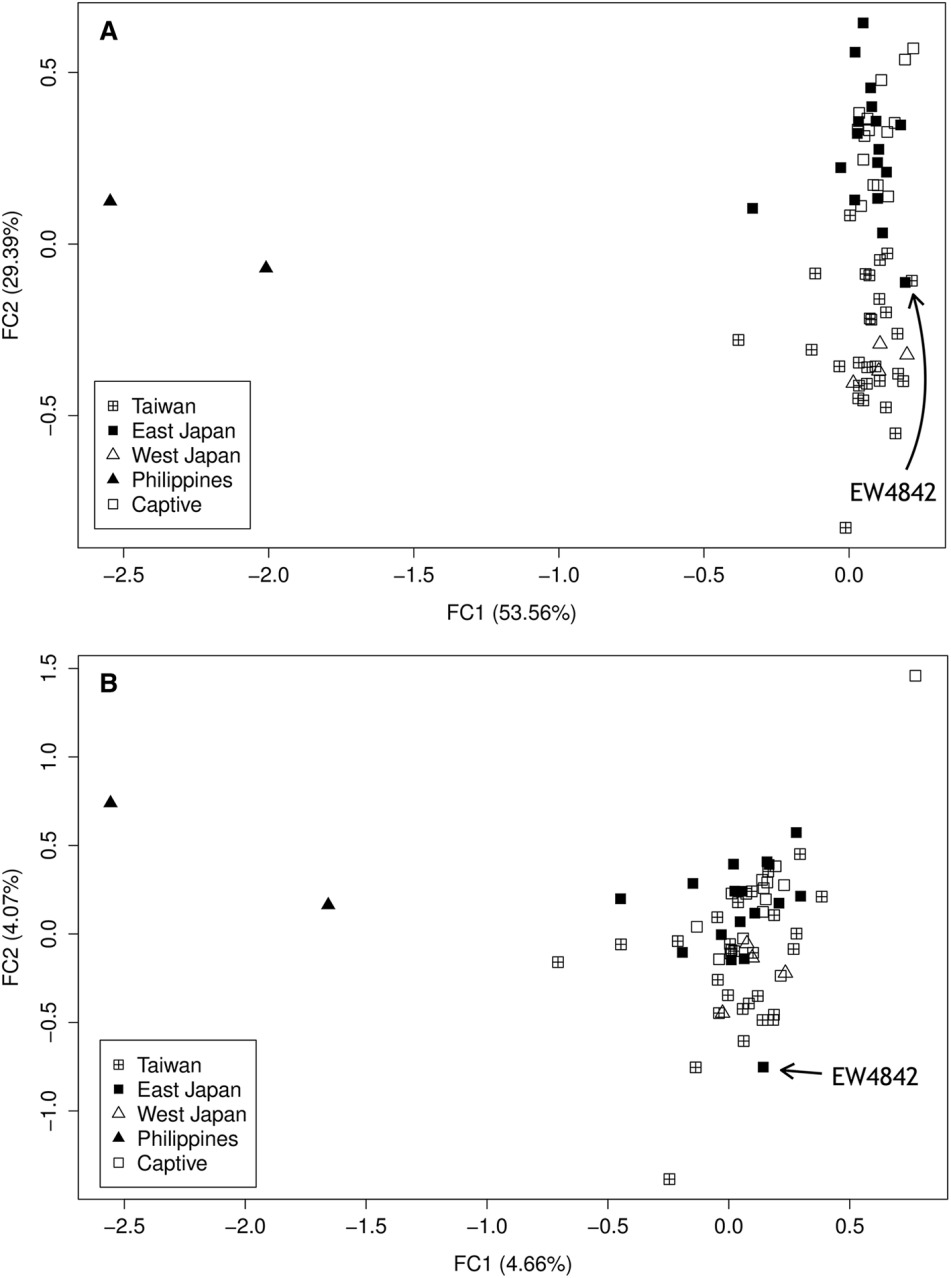
Results of the FCA for the CBD: **a** using the “sur population” option; **b** without using the “sur population” option. The two most informative factors (FC1 and FC2) were assigned as *X* and *Y* axes in the plot, and the *numbers* in parentheses in each* axis* indicate the percentage of the variance explained by the factor

For comparisons among CBD populations using the “sur population” option, all population-specific clusters could be identified and FC1 and FC2 accounted for 82.95% of the variance (Fig. 2a). Without using the option, the genotypes of the Philippine samples remained highly distinct, but other putative populations were less well resolved (Fig. 2b). The 14 captive dolphin samples provided by Japanese aquariums grouped with samples from East Japan rather than West Japan, while West Japan samples grouped with samples from Taiwan for FC1 vs. FC2. It is noteworthy that an East Japan sample, EW4842, was clustered with the Taiwan–West Japan samples, and the same clustering pattern was also found in the Geneland analysis (see below). This young male dolphin was stranded at the coast of Miyazaki, which was the most southerly sampling site for the putative East Japan population (Fig. 1). This “mis-grouping” could reflect limitations to the resolution of the analysis, evidence of direct migration between populations, or the result of a carcass drifting between regions (Bilgmann et al. 2011). The West Japan samples were segregated from the Taiwan samples and became an independent cluster by the third factor, FC3. This factor explained the remaining 17.05% of the variance (Fig. S2).

**Fig. 3:**
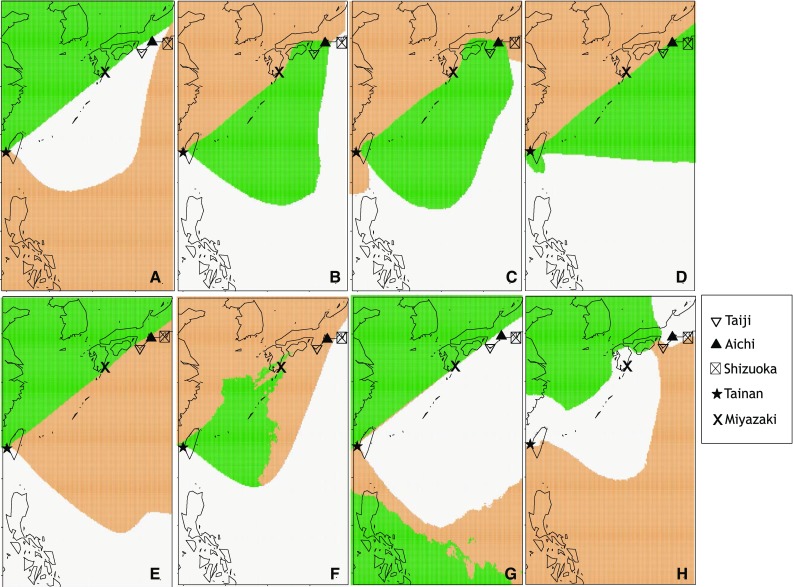
Eight variations of the individual population membership assignment patterns shown in the 10 runs with the highest LPP for *K* = 3 in Geneland analysis. The* colours* indicate the distribution of *K* clusters based on the mode of simulated posterior probability for each pixel. The landmarks mentioned in the text are shown

The ten simulations in the first step of the Geneland analysis all indicated the most likely number of populations for our sample set was *K* = 3. With the *K* fixed to *K* = 3 in the second step, the analysis suggested eight variations of population distribution patterns for CBD among the 10 runs with the highest LPP in 100 simulations. These eight variations all showed approximately the same clustering pattern, with a few samples showing inconsistent population membership (Fig. 3). The basic pattern was a cluster grouping samples from the west coast of Japan, west and north coast of Taiwan, and the sample collected in Miyazaki, Japan (“the West Coast Cluster”); a cluster for the samples from the east coast of Taiwan and from Taiji, Japan (“the East Coast Cluster”); and a cluster for the samples from the Philippines (“the South Tropical Cluster”). The samples collected from Tainan (southwestern Taiwan) and Shizuoka (eastern Japan) overlapped with all three clusters, but usually grouped with the South Tropical Cluster (Fig. 3a, b, f, h). The sample collected from Aichi (eastern Japan) grouped with the East Coast Cluster (Fig. 3a–e, g) and the West Coast Cluster (Fig. 3f, h).

### Population dynamics for CBD inferred from microsatellite analyses

To evaluate the population dynamics for CBD populations, we regrouped the samples into two clusters (for which sample sizes were deemed sufficient) based on the result of Geneland analysis: a West Coast Cluster (the samples from Miyazaki and the west coasts of Taiwan and Japan), and an East Coast Cluster (the samples from the east coast of Taiwan and Taiji). The samples from Tainan, Aichi, and Shizuoka were excluded due to the uncertainty of their population identity. *N*_e_ estimated from Migrate for the East Coast Cluster was slightly larger than for the West Coast Cluster (Table 2). *N*_e_ *N* ^−1^ for both populations was similar in magnitude, ranging from 0.042 to 0.059, or from 0.084 to 0.118, depending on what microsatellite mutation rate was used, and migration estimates suggested a higher rate from the West to the East Coast cluster (Table 2). The GeneClass analysis identified three potential first-generation migrants (Table S5). There was no support for sex-biased dispersal (Table S6).

**Table 2:** Estimates of effective population size times mutation rate (*N*_e_ *μ*) and number of migrants per generation (*N*_e_ *m*) from the two CBD populations recognized in Geneland analysis

	Source population	Host population			
		East coast		West coast	
*N*_e_ *μ*		0.400	(0.367–0.437)	0.321	(0.293–0.353)
*N*_e_ (low)		2000	(1836–2186)	1605	(1465–1766)
*N*_e_ (high)		4001	(3671–4371)	3211	(2930–3532)
*N*_e_ (low)/*N*		0.054	(0.05–0.059)	0.046	(0.042–0.05)
*N*_e_ (high)/*N*		0.108	(0.099–0.118)	0.092	(0.084–0.101)
*N*_e_ *m*	East coast			0.057	(0.046–0.070)
	West coast	0.106	(0.089–0.125)		

The *N*_e_ is calculated assuming that the average microsatellite mutation rate (*μ*) is 0.01% for *N*_e_ (high) and 0.02% for *N*_e_ (low). The ratio of effective to census population size (*N*_e_/*N*) is calculated using the census population size (*N*) estimated for the “Japanese Coastal” population (*N* = 37,000; Miyashita 1993) for the East Coast Cluster, and the *N* for the CBD in the southwestern Japanese waters (*N* = 35,000; Kasuya 2011) for West Coast Cluster. The 2.5th and 97.5th profile likelihood estimates are given in parentheses

### MtDNA genetic diversity of CBD and IPBD in the western and central North Pacific Ocean

We sequenced 42 CBD samples from Taiwan, East Japan, and Philippines, and seven IPBD samples from Taiwan and Japan. Together with the published sequences acquired from GenBank (*n* = 344), we used a 388 bp consensus mtDNA sequence from a total of 393 sequences and reconstructed five putative CBD populations (East Japan, Northeast China, Southeast China, Hawaiian Islands, and Palmyra Atoll) and four IPBD populations (Japan, Southeast China, South China, and Indonesia) (Fig. 1; Table S3). According to the AIC and BIC indices calculated by jModelTest, the best model for reconstructing a phylogenetic tree for the genus using our mtDNA sequences was HKY + I+G.

**Fig. 4:**
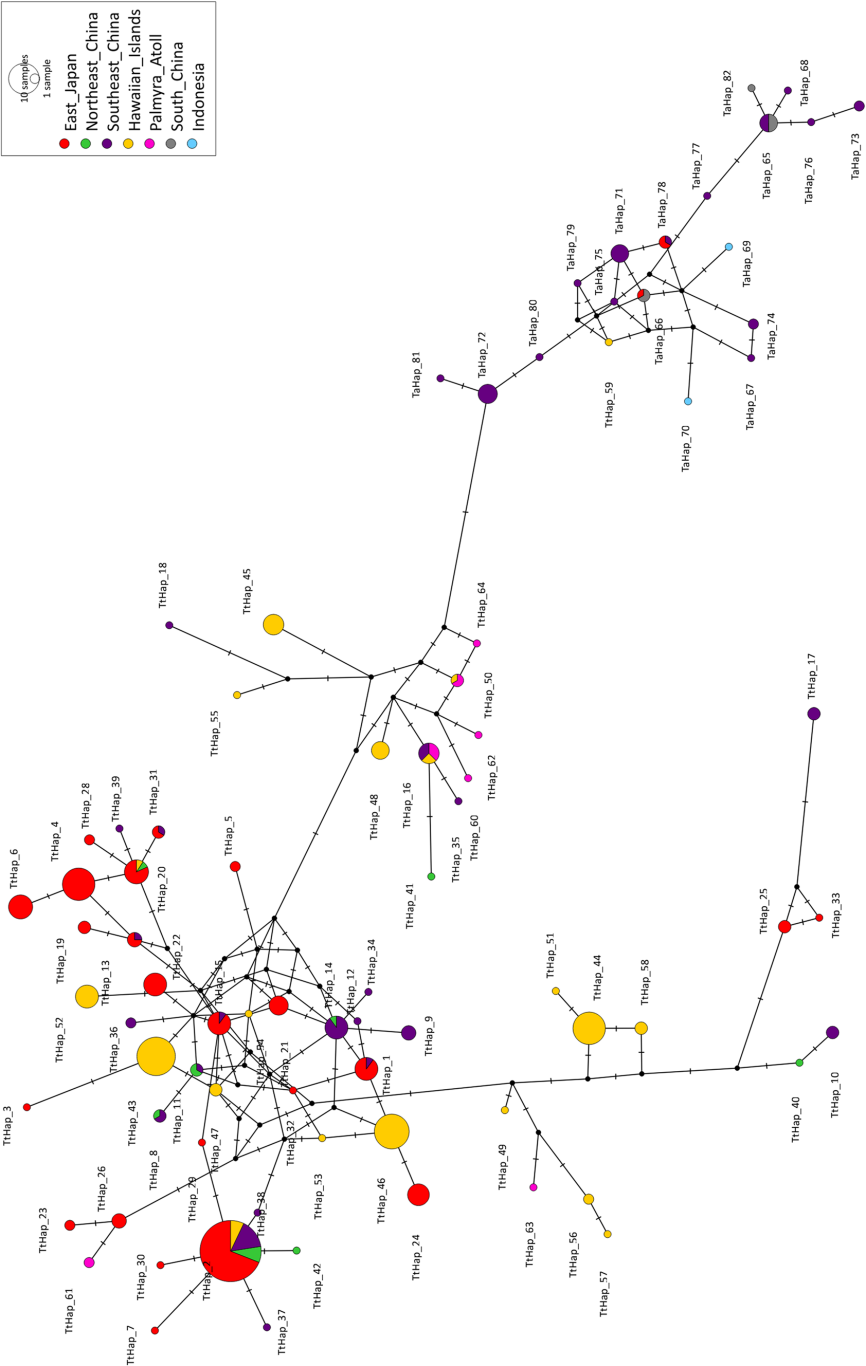
Median-joining network tree for CBD and IPBD mtDNA control region haplotypes. Each *circle* represents a unique haplotype. The *size of the circle* indicates the number of individuals having the haplotype and the *colour shade* indicates the proportion of each population within the haplotype. The *number* of hatch marks at the *lines* indicates the number of mutational steps separating the haplotypes. *Solid circles* indicate missing intermediate haplotypes

For CBD, we examined 353 sequences and identified 64 haplotypes defined by 82 variable sites, including two deletion gaps (Table S7). The overall haplotype diversity (*h*) was 0.935 and nucleotide diversity (*π*) was 0.0197. The *h* and *π* for each population are shown in Table 3. TtHap_2 was the most widespread haplotype, found in all populations except Palmyra Atoll (Fig. 4; Fig. S3; Table S8). It was also the dominant haplotype in the WNP (28.3% of all samples), where it was most common in Northeast China (42.9%, accession number AF459509-15), and in the school of dolphins culled in the drive fishery in 2005 (30.4%, previously published as Haplotype Ttr06, GenBank accession number AB303159). TtHap_16 was the only haplotype shared between the WNP (in Southeast China) and the tropical central Pacific (in Palmyra Atoll). It is noteworthy that in the phylogenetic tree, TtHap_17 (from southeast China), 25, and 33 (from eastern Japan) were isolated from the major CBD-IPBD lineage, potentially indicating that a lineage sorting process is still ongoing in CBD in the WNP populations (Fig. S3).

**Table 3:** Summary of the mtDNA haplotype diversity, nucleotide diversity, and indices for testing locus neutrality for the CBD and IPBD populations

	*n*	No. of haplotypes	Haplotype diversity (*h*)	Nucleotide diversity (*π*)	Tajima’s D	Fu’s Fs
CBD						
East Japan	160	23	0.870 (0.019)	0.01368 (0.00103)	−0.81835	−1.788
SE China	49	20	0.908 (0.025)	0.02193 (0.00314)	−0.74485	−1.607
NE China	14	8	0.824 (0.098)	0.01638 (0.00427)	−1.09647	0.216
Hawaii	119	20	0.868 (0.016)	0.02124 (0.00088)	−0.09236	1.449
Palmyra	11	7	0.909 (0.066)	0.01851 (0.00423)	−0.42215	0.526
Overall	**353**	**63**	**0.935 (0.008)**	**0.01966 (0.00079)**	**−1.25991**	−22.17**
IPBD						
Japan	3	2	0.667 (0.314)	0.00346 (0.00163)	NA	1.061
SE China	29	14	0.899 (0.036)	0.01365 (0.00110)	0.99207	−2.389
S China	6	3	0.733 (0.155)	0.01195 (0.00351)	0.99488	2.76
Indonesia	2	2	1 (0.5)	0.01039 (0.00519)	NA	1.386
Overall	**40**	**18**	**0.924 (0.022)**	**0.01395 (0.00084)**	**0.66414**	−4.005*

The SD for haplotype and nucleotide diversity is given in parentheses. Significant results (*p* < 0.05) are given in bold

* p < 0.05; ** p < 0.01

For IPBD, we identified 18 haplotypes defined by 19 variable sites in a total of 40 sequences from the coastal waters around Japan, Southeast China, and South China (GenBank accession numbers MF806001-18). The overall *h* was 0.924 and *π* was 0.014; the *h* and *π* of IPBD populations were lower than CBD populations in general, but samples sizes per population were small (Table 3). TtHap_59 was an IPBD haplotype from a putative CBD specimen collected from Hawaiian waters (M34066), and the introduction of this alien haplotype to the CBD population was regarded as a result of introgression in the distant past (Martien et al. 2012).

### Population structure and expansion history for CBD inferred from mtDNA data

Most pairwise *F*_ST_ and Φ_ST_ comparisons were statistically significant (Table 4). Fisher’s exact tests based on haplotype frequencies suggested that the five putative populations were well differentiated, except for the comparison between Northeast and Southeast China (Table S9). The clear differentiation between the Hawaiian Islands and Palmyra Atoll populations has been reported in the original paper (Martien et al. 2012); here, our analysis further reveals that Hawaiian Islands and Palmyra Atoll populations were also differentiated from the WNP populations. Within the WNP, the Northeast China population was the least differentiated, although the statistical insignificance could be largely due to deficient sample size, which was an issue for our Northeast China population sample.

**Table 4:** Pairwise mtDNA *F*_ST_ and φ_ST_ comparisons among the five putative CBD populations in the western-central North Pacific Ocean

	*n*	East Japan	SE China	NE China	Hawaii	Palmyra
East Japan	160		0.041**	0.020	0.114**	0.107**
SE China	49	0.080**		0.018	0.104**	0.076**
NE China	14	0.020	0.005		0.134**	0.135**
Hawaii	119	0.160**	0.059**	0.092**		0.109**
Palmyra	11	0.533**	0.304**	0.434**	0.243**	

The pairwise *F*_ST_ value is above the diagonal and the pairwise φ_ST_ value is below the diagonal

* p < 0.05; ** p < 0.01

A negative Tajima’s D was estimated for all putative populations, although none of the values were significantly different from zero (Table 3). A negative Fu’s Fs was estimated for the East Japan and Southeast China populations, but again, none of the estimates were statistically significant. The only exception was when all samples were pooled together, the Fu’s Fs estimate was negative and significantly different from zero (Table 3). The mismatch distributions for each putative population appeared to be multimodal (Fig. S4), even though fit to the expansion model could only be rejected for the Hawaiian Islands and Northeast China populations (Table S10).

### Interspecific comparisons

FCA analyses showed a clear genetic difference between CBD and IPBD (Fig. S5, S6). Using STRUCTURE, both Δ*K* and Ln*P*(*K*) values supported *K* = 2 (Table S11). The ancestry assignment test showed three CBD individuals, from Japan, Taiwan, and Philippines, respectively, that might have had an IPBD grandparent, although the probability was only between 7.1 and 11.9% (Fig. S6; Table S12).

## Discussion

### CBD population structure in the WNP

Miyashita (1993) suggested the CBD in the WNP is mainly distributed in 30°–42°N and west of 160°E, with a density gap at 142°–145°E as a boundary separating the “Japanese coastal” population (west of 142°E) and “Japanese offshore” population (east of 145°E). That boundary is tentatively supported by a telemetry study showing that the CBD population targeted by the Japanese coastal drive fishery (Kishiro and Kasuya 1993) is unlikely to utilise the waters further than 200 nautical miles from land (Tanaka 1987). Since most of the samples were collected from the dolphins caught in the coastal drive fishery, our East Japan sample very likely represents this Japanese coastal population. Our Geneland analysis further suggests that the range of this Japanese coastal population could be extended further south to the eastern coast of Taiwan (22°–25°N, east of 121°E), and we, therefore, call this the “East Coast Cluster” to avoid confusion. The east coasts of Taiwan and Japan (between 22° and 42°N) are together embedded in a unique oceanic biogeographic province, of which the main characteristic is sharing the speedy, warm, relatively high saline Kuroshio current flowing northeastward from Luzon in the Philippines to the east coast of Japan year-round (Wyrtki 1975; Spalding et al. 2012; Fig. 1). Despite the uncertainty of the habitat preference for this East Coast Cluster CBD population, it is very likely that the strong, constant Kuroshio Current plays a crucial role in defining their habitat (Tanaka 1987). Similar structure of connectivity along the east coasts of Taiwan and Japan is also seen in short-finned pilot whales (*Globicephala macrorhynchus*) (Chen et al. 2014), Risso’s dolphins (*Grampus griseus*), and Fraser’s dolphins (*Lagenodelphis hosei*) (Chen 2016). However, we acknowledge that further fine-scale population sub-division within the Cluster is possible, as it seems to be a common pattern for CBD elsewhere in the world (e.g., Mirimin et al. 2011; Martien et al. 2012; Richards et al. 2013; Gaspari et al. 2015a), but our sample size for eastern Taiwan was too small (*n* = 4) to reveal such pattern, if it does exist. In contrast to some earlier studies (e.g., Wiszniewski et al. 2010), we found no evidence for sex-biased dispersal, though we cannot rule this out as the test has relatively low power (Goudet 2002).

On the other hand, Kasuya et al. (1997) found a subtle difference in several life history traits (e.g., the body length at sexual maturity, the age of sexual maturity, and the interval of breeding) between CBD caught in Taiji (eastern Japan) and Iki (southwestern Japan), suggesting that CBD populations between the east and west coasts of Japan could be differentiated (cited in Kasuya 2011). This hypothesis is tentatively supported by Hayano (2013), who studied a 520 bp mtDNA control region sequences in 42 CBD from the east and west coasts of Japan and found that seven of the ten samples collected from the west coast were grouped in a unique phylogenetic cluster with a bootstrap support value of 71%. Our FCA and Geneland results also support the differentiation of CBD populations between the west and east coasts of Japan (i.e., between the Sea of Japan and the Pacific coast of Japan), although the sample size from the population west of Japan is too small for robust inference. This pattern has been reported for other cetaceans found in the same region, e.g., in minke whales (*Balaenoptera acutorostrata*; Abe et al. 2000) and Dall’s porpoises (*Phocoenoides dalli*; Hayano et al. 2003).

Our results further reveal that there may be another coastal CBD population in the vicinity of the Taiwan Strait (western Taiwan), although its relationship with the CBD population from West Japan (in the Sea of Japan) is ambiguous, and so we grouped the samples together as a West Coast Cluster. Earlier studies for bottlenose dolphins in the coastal region of WNP mainly focused on the ecological, morphometric, and genetic differences between CBD and IPBD (Gao et al. 1995; Wang et al. 1999, 2000; Yang et al. 2005; Kurihara and Oda 2007), providing limited insight into the population differentiation within the CBD species. Our study highlights the need for further careful investigations into CBD in this region, including the distribution, habitat preferences, or behaviours of the dolphins, to shed more light on the evolutionary mechanisms driving the CBD populations in the Asian coastal waters to differentiate.

Our FCA and Geneland results suggest a fairly distinct population of CBD inhabiting Philippine waters, though the sample size is too small for strong inference. One of the samples could possibly be a hybrid with other delphinid species; however, we have insufficient data to be precise about which one. Dolar et al. (2006) reported that the CBD population in central Philippine waters (Sulu Sea and the Tañon Strait) was found exclusively in shallow and intermediate waters inside of the shelf break, and that this preference may limit the dispersal of the Philippine population. Although various lines of evidence indicate that possibility, more data are needed to resolve this question.

### Population dynamics of the CBD in the WNP

The *N*_*e*_ estimated for the West Coast Cluster and the East Coast Cluster are only about a quarter to a tenth of the *N*_e_ estimated for the more pelagic central Pacific populations (Martien et al. 2012). This agrees with an earlier report that suggests that the *N*_*e*_ for coastal CBD populations tends to be smaller than for pelagic populations (Louis et al. 2014a). Our calculation showed the *N*_e_ *N* ^−1^ estimates for both West and East Coast Clusters are similar in magnitude, ranging between 0.042 and 0.118. This range is consistent with estimates proposed by meta-analysis of *N*_e_ *N* ^−1^for wildlife populations (Frankham 1995).

Tajima’s D and Fu’s Fs estimates were statistically insignificant and the mismatch distributions appeared multimodal in both demographic and spatial models (though not always significantly different from the model for expansion). In general, there was no strong evidence for population expansion.

### Possible mechanisms that shape the CBD population structure in the WNP

The migrate analysis suggests that long-term gene flow between the East and West Coast Clusters is limited to less than one migrant per generation. On the other hand, the GeneClass analysis identified three contemporary first-generation migrants, suggesting the presence of ongoing gene flow between the two populations. Low levels of gene flow have been reported between the coastal and pelagic populations in the eastern North Atlantic Ocean (Louis et al. 2014a), and among the regional populations around the Hawaiian Islands (Martien et al. 2012). It has been proposed that this could be promoted by assortative mating due to the constrained preference of natal habitat, specialised diet, and possibly culture familiarity (Hoelzel et al. 1998; Möller et al. 2007; Cantor and Whitehead 2013; Louis et al. 2014a, b). In our case, although the strong correspondence between the population structure and contrasting oceanographic features (i.e., shallow continental shelves vs. sharp continental slopes) implies population-specific resource preference, the structure may have been promoted by historic isolation (possibly during the glacial period). We propose that the Kuroshio current could play an important role in “regulating” the frequency of gene exchange.

During the glacial period, the influence of the Kuroshio current on the coasts of the eastern Asian continent was weakened as the flows to the East China Sea and South China Sea were limited (Ijiri et al. 2005; Jiang et al. 2006). The Tsushima Warm Current, a branch of Kuroshio Current carrying warm water into the Sea of Japan through the Tsushima Strait, was suspended during the Last Glacial Maximum (Itaki et al. 2004). Therefore, the oceanography of the region may not have promoted connectivity during the glacial period as much as today, and the lack of warmer water introduced by the Kuroshio Current from the south could have generated more contrasting physical conditions between the shallower western coastal and the deep eastern continental slope habitats. This habitat distinction may have also reinforced the reduction of connectivity between the two populations during that period. In contrast, today, the current itself and its branch currents constantly drive the surface waters in and out the shallow coastal region (Cho et al. 2009; Matsuno et al. 2009; Jan et al. 2010), and it has been observed that the movement of CBD can be influenced by the flow of Kuroshio Current (Tanaka 1987). Our data suggest recent migration between these regions, which may reflect this environmental facilitation (though the populations remain differentiated).

### Sympatric relationship between IPBD and CBD

Our mtDNA data agree with the previous studies showing clear phylogenetic differentiation between IPBD and CBD (Wang et al. 1999; Kakuda et al. 2002; Natoli et al. 2004; Yang et al. 2005; Kita et al. 2013; Moura et al. 2013). Our microsatellite data also exhibit a distinct difference between IPBD and CBD. The microsatellite data also provide some indication of limited gene flow between these two species, indicating three individuals that might have hybrid ancestry. The two species are known to have interbred and produced reproductively viable female hybrids in a captive environment (Hale et al. 2000), and potential descendants of hybrids between the two species are found in the CBD populations in Hawaiian and Japanese waters (Martien et al. 2012; Hayano 2013). However, we cannot eliminate the possibility that the interbreeding was between CBD and other delphinid species that are closely related to IPBD but live sympatrically with the CBD, such as pantropical spotted dolphin (*Stenella attenuata*), striped dolphin (*Stenella coeruleoalba*), spinner dolphin (*Stenella longirostris*), common dolphin (*Delphinus* sp.), or Fraser’s dolphin (LeDuc et al. 1999; Kingston et al. 2009; Möller et al. 2008; McGowen 2011; Amaral et al. 2012). This has the potential to produce misleading results when the signal for hybridisation is weak, as in the case of our study. In fact, among four potential hybridisations between the *Tursiops* congeneric species (three from this study and one from Martien et al. 2012), one of the putative hybrid animals was similar in appearance to Fraser’s dolphin, and two (one form eastern Taiwan and the other from Hawaiian Islands) were sampled from a region where the occurrence of IPBD has never been documented (Yang et al. 1999; Chou 2007; Baird et al. 2013). Since there is evidence of polyphyly among the *Tursiops*–*Stenella*–*Delphinius* complex of species (LeDuc et al. 1999; Kingston et al. 2009; McGowen 2011; Amaral et al. 2012), the evidence for hybridisation, therefore, needs to be interpreted carefully.

Our genetic data for IPBD were obtained from three putative aggregation sites in Taiwanese and Japanese waters: Amakusa (southwestern Japan), Mikura Islands (southeastern Japan), and western Taiwan; and the FCA result showed distinct clustering for those samples (Fig. S5). The microsatellite data are consistent with the population structure proposed based on mtDNA data (Hayano 2013), but to verify the hypothesis of IPBD population structure in these waters, further examination using more samples from the same and further sites is necessary.

### Conservation implications

Our results indicate that there are at least two populations of CBD distributed parapatrically in the coastal waters around Taiwan and Japan, corresponding to the distribution of shallow continental shelf or deep continental slope habitats. Further sampling may well reveal further structure in the broader region. Although our analyses detected some recent immigration, the long-term estimates show limited gene flow between the two populations. This potentially agrees with earlier analyses that show that habitat specialisation plays an important role in differentiating inshore and offshore populations (Hoelzel et al. 1998; Möller et al. 2007; Louis et al. 2014b; Gaspari et al. 2015b). The two CBD populations are likely affected by different anthropogenic threats. For instance, the small-scale dolphin drive fishery appears to target primarily the East Coast Cluster CBD (see Kasuya 2011). On the west coast, habitat loss and degradation, pollution, acoustic disturbances, and fisheries interactions have been identified as risks for coastal cetacean species (Perrin et al. 2005; Jefferson et al. 2009; Choi et al. 2013; Slooten et al. 2013). It is, therefore, justifiable to manage them as separate CBD populations, and further investigations are needed to evaluate their resistance to those threats. We address our aim to improve understanding of the pattern and origin of population structure in this region by identifying previously unrecognised boundaries and illustrating the historical and environmental contexts. This contributes both towards the more effective conservation of species in this genus, and towards a better understanding of processes associated with population differentiation in this region.

## Electronic supplementary material

Below is the link to the electronic supplementary material.

Supplementary material 1 (PDF 526 kb)

Supplementary material 2 (XLSX 77 kb)
