# Supplementary material for: Genetic diversity of bottlenose dolphin (Tursiops sp.) populations in the western North Pacific and the conservation implications
Source: Mar Biol. 2017 Sep 11;164(10):202. doi: 10.1007/s00227-017-3232-8 (PMC5592193; doi:10.1007/s00227-017-3232-8)

Genetic diversity of bottlenose dolphin (*Tursiops sp.*) populations in the western North Pacific and the conservation implications

Ing Chen[1,2], Shin Nishida[3], Wei-Cheng Yang[4], Tomohiko Isobe[5], Yuko Tajima[6], A. Rus Hoelzel[1]

1 School of Biological and Biomedical Sciences, University of Durham, South Road, Durham, DH1 3LE, United Kingdom

2 Department of Life Sciences, National Cheng Kung University, 1 Da-Xue Road, East District, Tainan, 70101, Taiwan

3 Science Education, Faculty of Education and Culture, University of Miyazaki, 1-1 Gakuen-Kibanadai-Nishi, Miyazaki, 889-2192, Japan

4 Department of Veterinary Medicine, National Chiayi University, 580 Xinmin Road, Chiayi, 60054, Taiwan

5 National Institute for Environmental Studies, 16-2 Onogawa, Tsukuba, 305-8506, Japan

6 Division of Vertebrate, Department of Zoology, National Museum of Nature and Science, 4–1–1 Amakubo, Tsukuba-shi, Ibaraki, 305-0005, Japan

Supplementary figures

Fig.S1.

Individual's population membership under a series of K predicated by STRUCTURE analysis using 20 microsatellite loci data and the LOCPRIOR option for K=2. Each column represents one individual, and the colour portion in each column indicated the probability of the individual being assigned to a population.

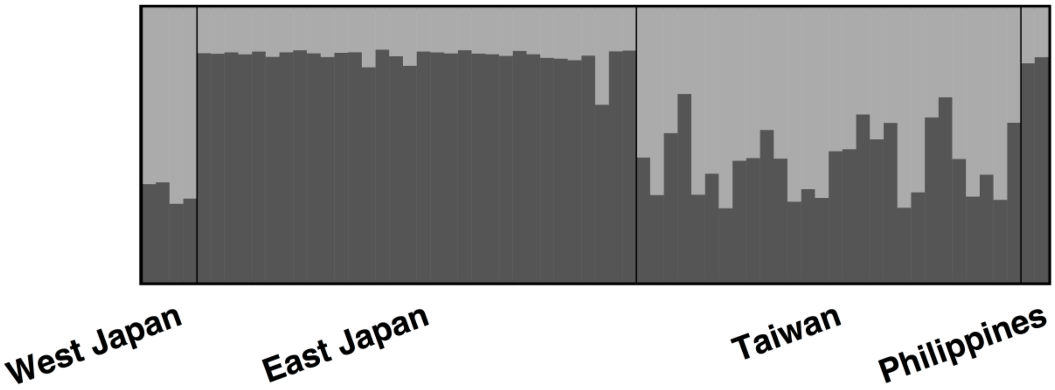

Fig. S2.

The results of the FCA for the CBD using the “sur population” option with the first and third most informative factors (FC1 and FC3) as the X and Y axes of the plot. The numbers in parentheses in each axis indicates the percentage of the variance explained by the factor.

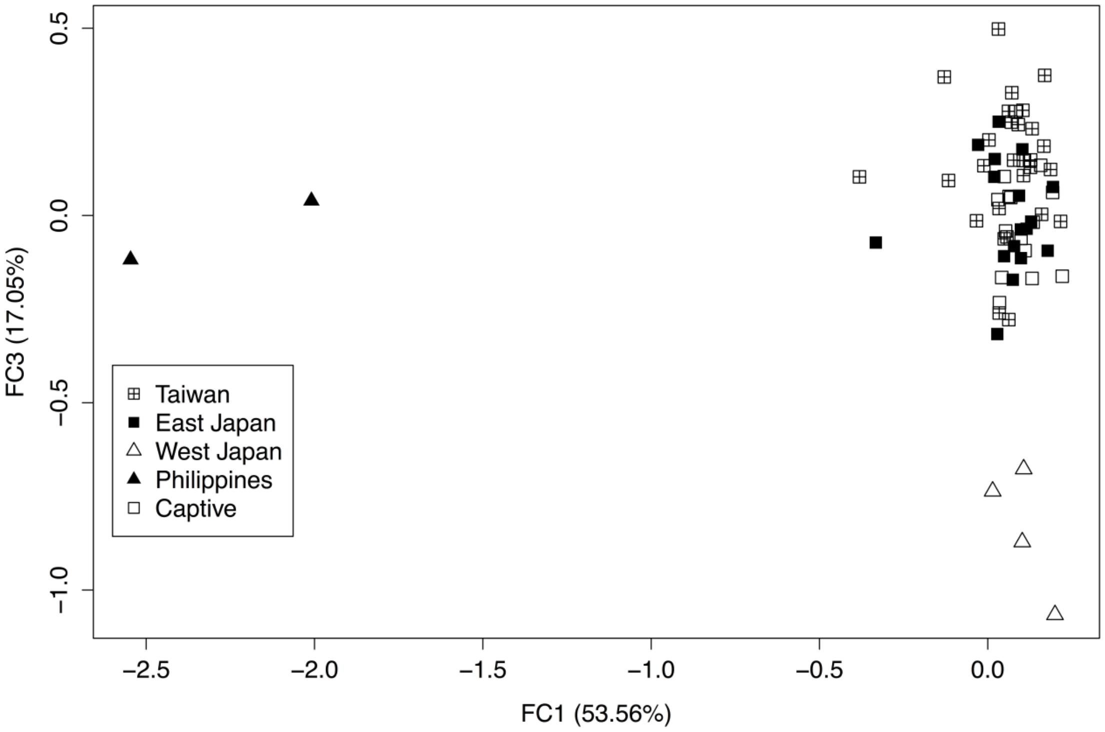

Fig. S3.

Phylogenetic relationship of the mtDNA haplotypes for the CBD and IPBD from the western and central North Pacific Ocean. The number at the branch indicates the bootstrap probability (%); those smaller than 50% are not shown.

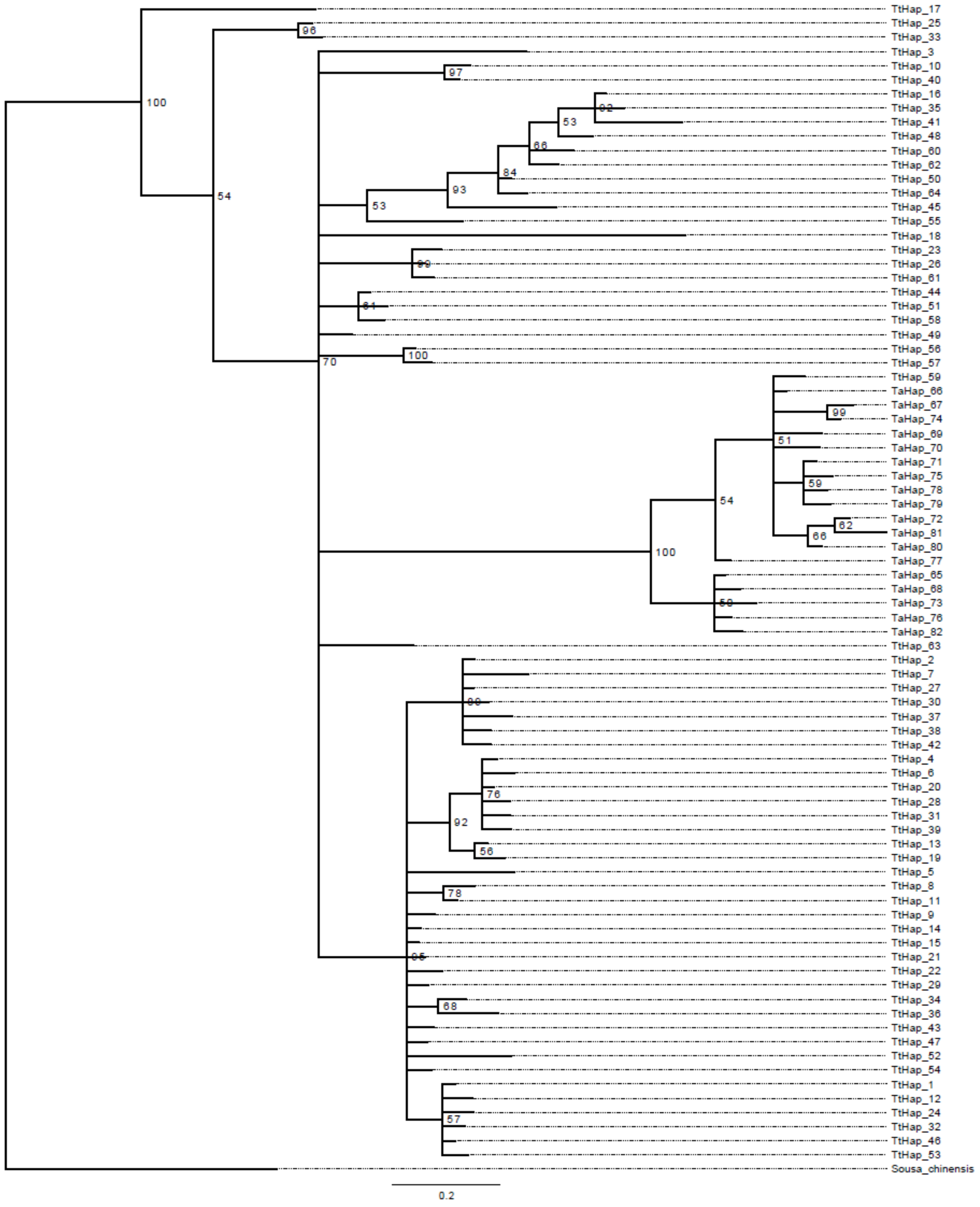

Fig. S4.

Observed and expected mismatch distributions for the CBD populations in the western and central North Pacific Ocean under the demographic (A) and spatial expansion models (B). The vertical bars (in grey) in the panels indicate the model frequency in each scenario.

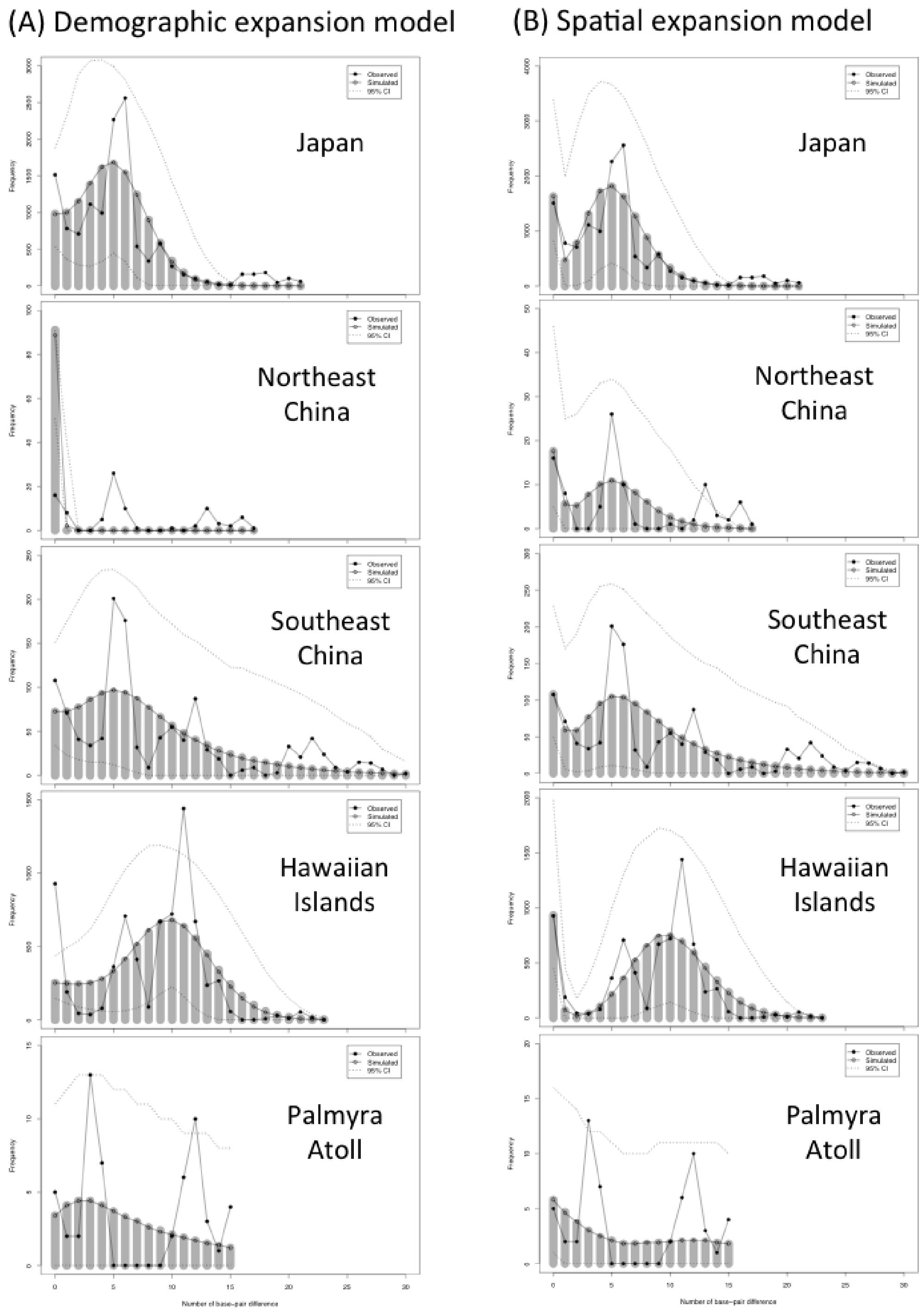

Fig. S5.

The result of a factor correspondence analysis (FCA) for all acquired bottlenose dolphin samples (CBD: common bottlenose dolphin; IPBD: Indo-Pacific bottlenose dolphin), without using the “sur population” option. Numbers in parentheses indicates the percentage of the variance explained by the factor/axis.

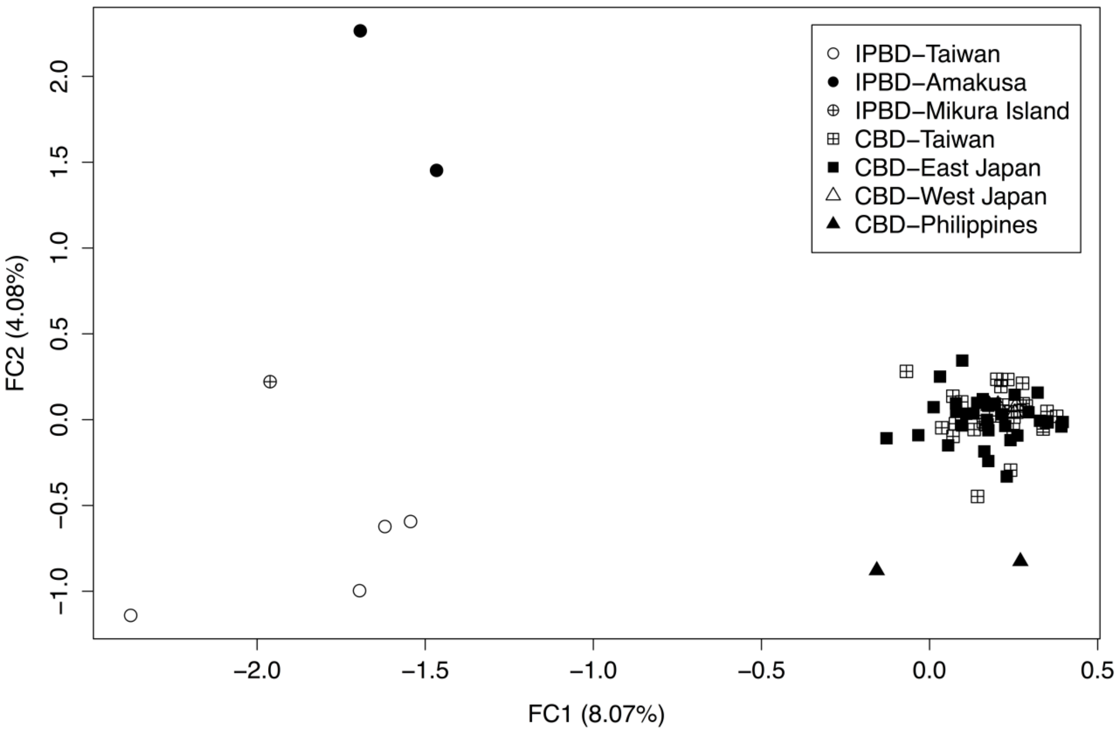

Fig. S6.

The best population model (K=2) predicated by STRUCTURE analysis for CBD and IPBD. Each column represented one individual, and the colour portion in each column indicated the probability of the individual being assigned to a population. The arrows indicate the three potential descents of hybrids between the two species (for further information, see supplementary Table S5).

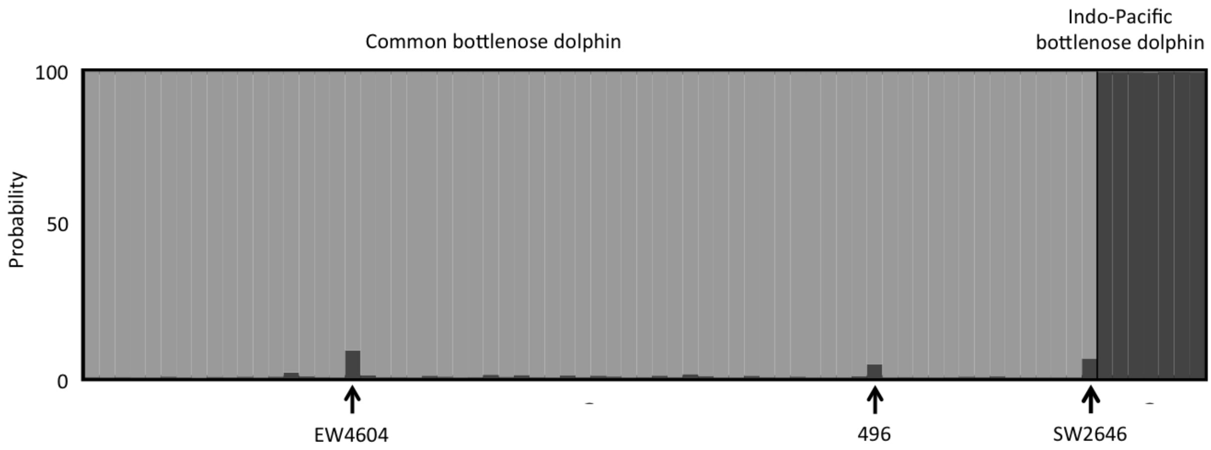

Supplement: Supplementary file 1 — Supplementary material 1 (PDF 526 kb) [file 227_2017_3232_MOESM1_ESM.pdf]
